# Radiology of Fractures in Intoxicated Emergency Department Patients: Locations, Mechanisms, Presentation, and Initial Interpretation Accuracy

**DOI:** 10.1097/MD.0000000000000980

**Published:** 2015-06-19

**Authors:** Yuka Morita, Taiki Nozaki, Jay Starkey, Yuka Okajima, Sachiko Ohde, Masaki Matsusako, Hiroshi Yoshioka, Yukihisa Saida, Yasuyuki Kurihara

**Affiliations:** From Department of Radiology, St. Luke's International Hospital, Chuo-ku, Tokyo, Japan (YM, TN, JS, YO, MM, YS, YK); Department of Radiological Sciences, University of California Irvine, Irvine, California (TN, HY); Department of Radiology, University of California San Francisco, San Francisco, California (JS); and Center for Clinical Epidemiology, St. Luke's Life Science Institute, St. Luke's International Hospital, Chuo-ku, Tokyo, Japan (SO).

## Abstract

The purpose of this study was to investigate the relationship of alcohol intoxication to time-to-presentation following injury, fracture type, mechanism of injury leading to fracture, and initial diagnostic radiology interpretation performance of emergency physicians versus diagnostic radiologists in patients who present to the emergency department (ED) and are subsequently diagnosed with fracture.

Medical records of 1286 patients who presented to the ED and were diagnosed with fracture who also underwent plain film or computed tomography (CT) imaging were retrospectively reviewed. The subjects were divided into intoxicated and sober groups. Patient characteristics, injury-to-presentation time, fracture location, and discrepancies between initial clinical and radiological evaluations were compared.

Of 1286 subjects, 181 patients were included in the intoxicated group. Only intoxicated patients presented with head/neck fractures more than 24 hours after injury. The intoxicated group showed a higher rate of head/neck fractures (skull 23.2% vs 5.8%, face and orbit 30.4% vs 9.5%; *P* < 0.001) and a lower rate of extremity injuries. The rate of nondiagnosis of fractures by emergency physicians later identified by radiologists was the same in both groups (7.7% vs 7.7%, *P* = 0.984).

While the same proportion of intoxicated patients presented more than 24 hours following injury, only intoxicated patients presented with craniofacial and cervical spinal fractures during this period. Alcohol-related injuries are more often associated with head/neck fractures but less extremity injuries. The rate of fractures missed by emergency physicians but later diagnosed by radiologists was the same in intoxicated and sober patients.

## INTRODUCTION

Alcohol intoxication has been related to a variety of acute and chronic medical conditions including traumatic injuries, across all age groups and settings from urban to rural.^1–6^ Intoxication-related trauma is a leading cause of emergency department (ED) visits, including motor vehicle accidents, falls, assaults, and other types of trauma. In the United States, an estimated 620,000 intoxicated patients are evaluated in the ED each year,^7^ and ED visits attributable to intoxication are on the rise.^8^ Initial assessment of intoxicated patients can be challenging due to their altered mental status. Additionally, initial interpretation of plain radiographs is generally performed by emergency physicians rather than radiologists in many hospitals in Japan.

Past research results suggest intoxication-related trauma is associated with higher rates of traumatic brain injury, morbidity, mortality,^1–6^ and computed tomography (CT) and magnetic resonance (MR) imaging utilization.^9–10^ However, few studies have focused on the relationship between delayed time-from-injury to presentation to the ED,^11^ intoxication and skeletal fracture locations,^12^ mechanism of injury leading to fracture,^12^ or accuracy of initial radiographic interpretations by nonradiologists.^13^

We hypothesized (1) more intoxicated patients with fractures will present to the ED greater than 24 hours after injury than sober patients, (2) intoxicated patients treated in the ED are at higher risk of craniofacial and cervical spine fractures compared to sober patients, (3) the mechanism of injury (eg, injury from traffic accidents, falls, assault, etc.) is different in intoxicated versus sober patients, and (4) emergency physicians miss fractures in intoxicated patients more often due to unreliable history and physical examination.

Therefore, the purpose of our study was to investigate the relationship of alcohol intoxication to time-to-presentation following injury, fracture type, mechanism of injury leading to fracture, and incorrect initial diagnostic radiographic interpretation by emergency physicians.

## MATERIALS AND METHODS

### Patients

This single-institution retrospective study was approved by our institutional review board. Patient informed consent was waived.

Our study was conducted by radiologic and chart review, and included adult patients over the age of 20 who were treated in our ED and underwent radiography (n = 13,752) or CT (n = 11,545) from July 2010 to December 2011. The study population was further limited to those patients diagnosed by diagnostic imaging with fracture by radiologist interpretation (n = 1286). In total, the study included 748 males and 538 females with a median age of 57.0 years (range, 20–102 years).

### Methods

We identified all ED patients with fractures diagnosed by plain film radiography or CT via query of the electronic medical record and reporting system of the department of radiology. We determined if fracture was related to alcohol intoxication based on the visit record indicating either a diagnosis of “alcohol intoxication” or a chief complaint containing text with any variation of “alcohol.” Patients were then divided into two groups: intoxicated and sober. When we could not determine intoxication status from the chart, patients were placed into the sober group.

Patient characteristics, injury-to-presentation time (grouped by within 24 hours or after 24 hours), mechanisms of injury (including traffic-related categories, falls from standing, intermediate heights of ≤20 feet, and high heights >20 feet, and assault), and fracture locations were also ascertained via chart review.

We subsequently reviewed radiologic studies and categorized the location of injury by region (including head, cervical spine, thoracolumbar spine, thorax, pelvis, upper limb, and lower limb). All studies were reviewed by two radiologists in consensus, including an experienced musculoskeletal board-certified radiologist and a radiology resident.

To assess discrepancies between initial clinical and radiological interpretations by emergency physicians and the final radiologist interpretation, we compared the ED notes with final radiology reports. An experienced musculoskeletal board-certified radiologist and a radiology resident then reviewed cases with discrepancies and by consensus determined whether a major discrepancy had occurred, defined as a missed fracture that necessitated a change in clinical management such as a case requiring operation or cast fixation.

### Statistical Analysis

All statistical analyses were performed by a statistician using SPSS version 20.0 (IBM, Armonk, NY). Comparison of categorical data was made using the chi-square test, and the Mann–Whitney *U* test was used to compare time to treatment over 24 hours after injury between the intoxicated and sober groups. A *P*-value of less than 0.05 was considered statistically significant.

## RESULTS

### Patient Characteristics

Of 1286 subjects included in our study, 181 patients were included in the intoxicated group (148 males, 33 females, mean age 51.5 ± 16.1, range 20–85) and 1105 patients in the sober group (600 males, 505 females, mean age 57.0 ± 20.7, range 20–102). The mean age of the intoxicated group was significantly lower than sober group (*P* < 0.001). In the intoxicated group, the proportion of males was higher than in the sober group (81.8% vs 54.3%, *P* < 0.001; Table 1).

**TABLE 1:**
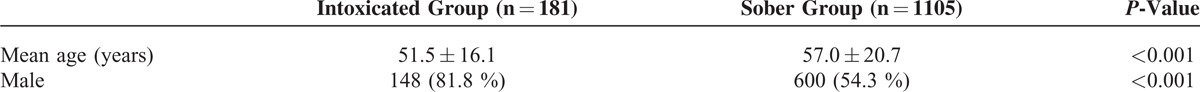
Patient Characteristics

### Time-of-Injury to Presentation

There were no significant differences in the proportion of patients who presented to the ED over 24 hours after injury in the intoxicated and sober groups overall (9.9% vs 11.7%, *P* = 0.498). Of the patients who presented to the ED over 24 hours after injury, mean days to presentation in intoxicated group were shorter than in the sober group (1.67 ± 1.53 vs 3.43 ± 4.01, *P* < 0.05). However, there were no patients in the sober group who presented late to the ED with skull and cervical spine fractures, while 4 out of 18 (22.0%) patients in the intoxicated group had such fractures (Table 2).

**TABLE 2:**
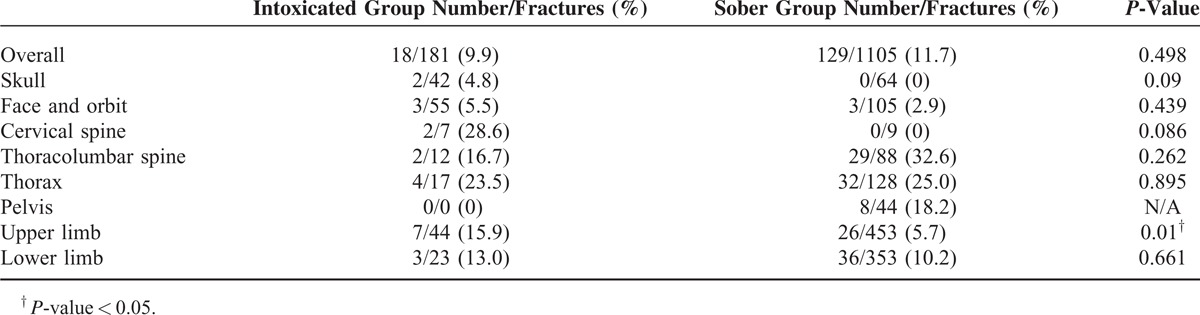
Time-to-Presentation (>24 hours)

### Fracture Locations

The intoxicated group showed a higher rate of head/neck fractures (skull 23.2% vs 5.8%; *P* < 0.001, face and orbit 30.4% vs 9.5%; *P* < 0.001, cervical spine 3.8% vs 0.8%; *P* < 0.001) and a lower rate of extremity injuries (upper limb 24.3% vs 41.0%; *P* < 0.001, lower limb12.7% vs 31.9%; *P* < 0.001) compared to the sober group (Table 3).

**TABLE 3:**
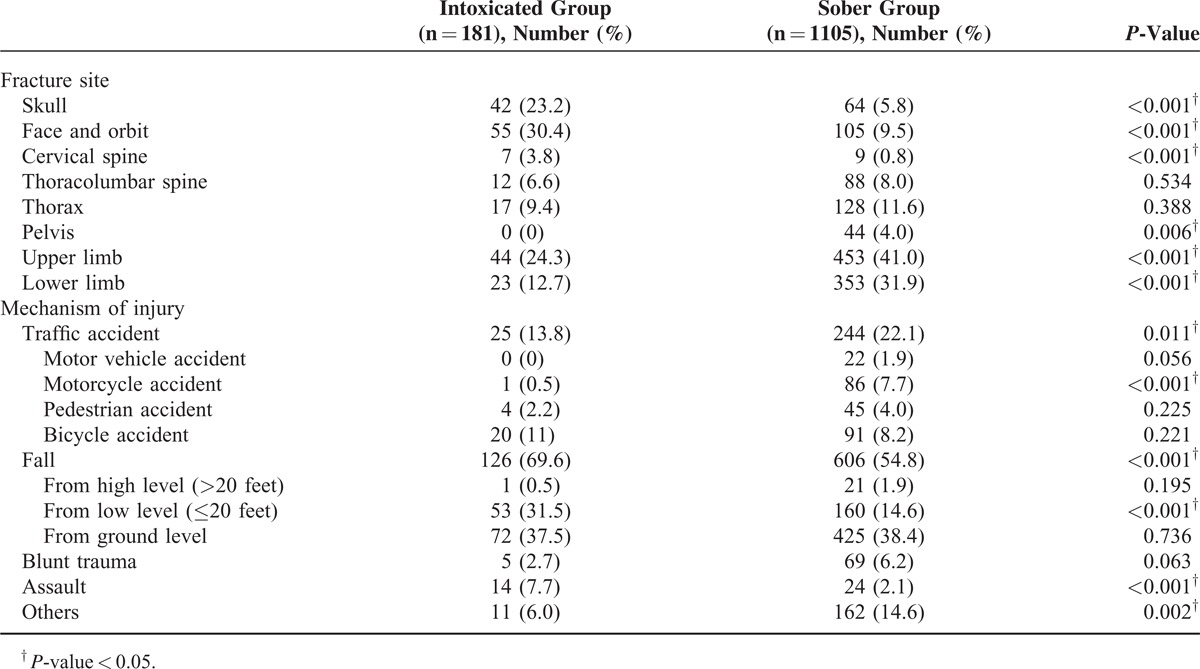
Fracture Site and Mechanism of Injury

### Fracture Mechanisms

Compared to the sober group, a larger proportion of patients were injured due to falls (69.6% vs 54.8%, *P* < 0.001), especially from intermediate heights (31.5% vs 14.6%, *P* < 0.001). More intoxicated patients were injured by assault (7.7% vs 2.1%, *P* < 0.001). A smaller proportion of patients in the intoxicated group were injured in motor vehicle accidents (13.8% vs 22.1%, *P* = 0.011). There was no difference in the rate of bicycle accidents (8.2% vs 11.0%, *P* = 0.221). Injury by blunt trauma was less frequent in the intoxicated group than in the sober group (2.7% vs 6.2%, *P* = 0.063), though this difference was not statistically significant (Table 3).

### Initial Radiologic Interpretation

There was no significant difference between fractures missed on initial interpretation by emergency physicians that qualified as major discrepancies in intoxicated and sober patients (7.7% vs 7.7%, *P* = 0.984). When we evaluated the rate of missed fractures divided into each body region, there were no significant differences between groups.

## DISCUSSION

### Time-to-Presentation

**FIGURE 1:**
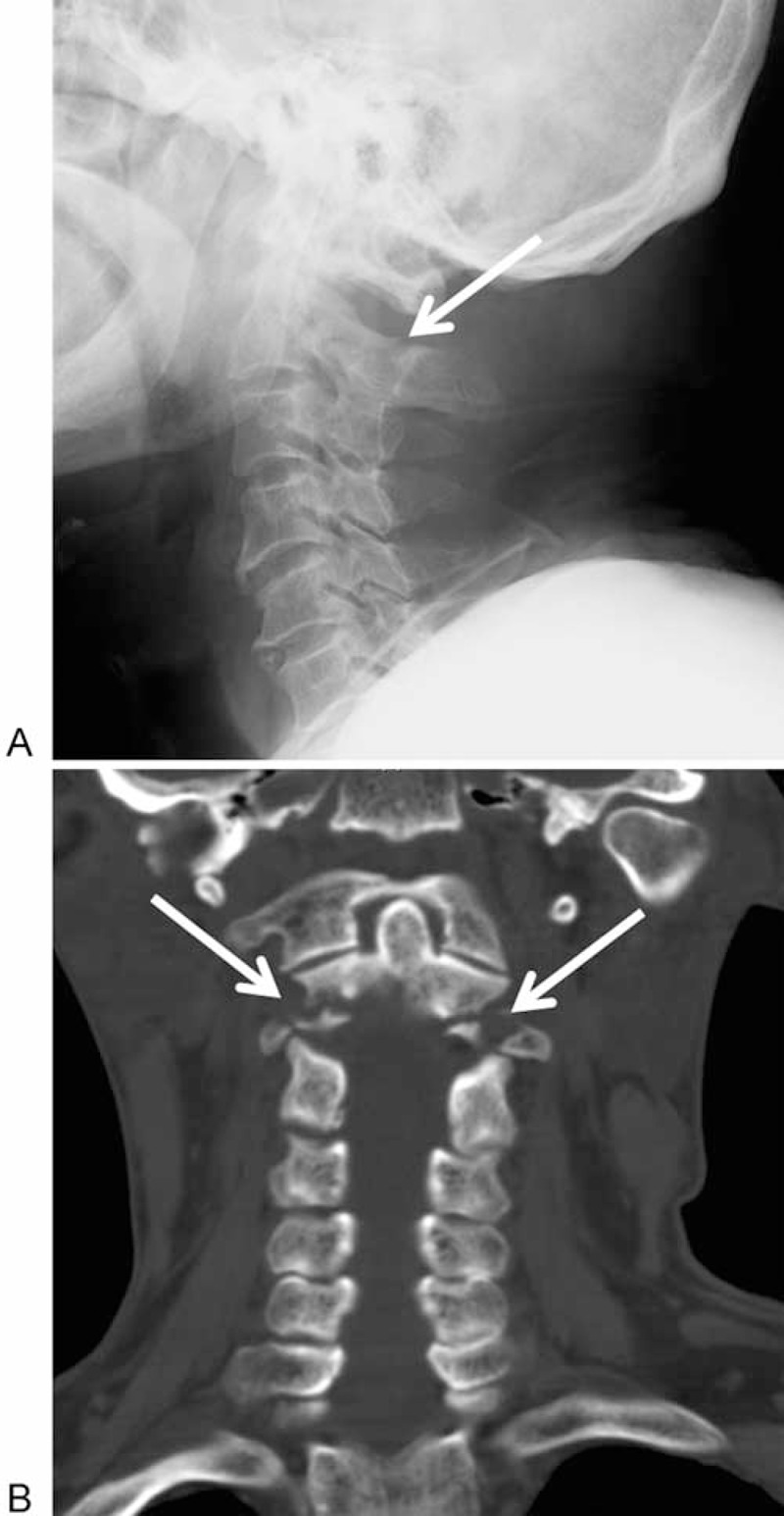
Plain radiograph and CT of the cervical spine in a 67-year-old male with cervical fracture (C2–C4). He fell down stairs while intoxicated. Two days later, he visited our ED with a chief complaint of neck pain. (A) Lateral plain radiograph of the cervical spine shows a fracture through the lamina of the atlas (arrow). (B) Coronal CT shows fractures through both sides of the laminae and arch of the atlas (arrow).

**FIGURE 2:**
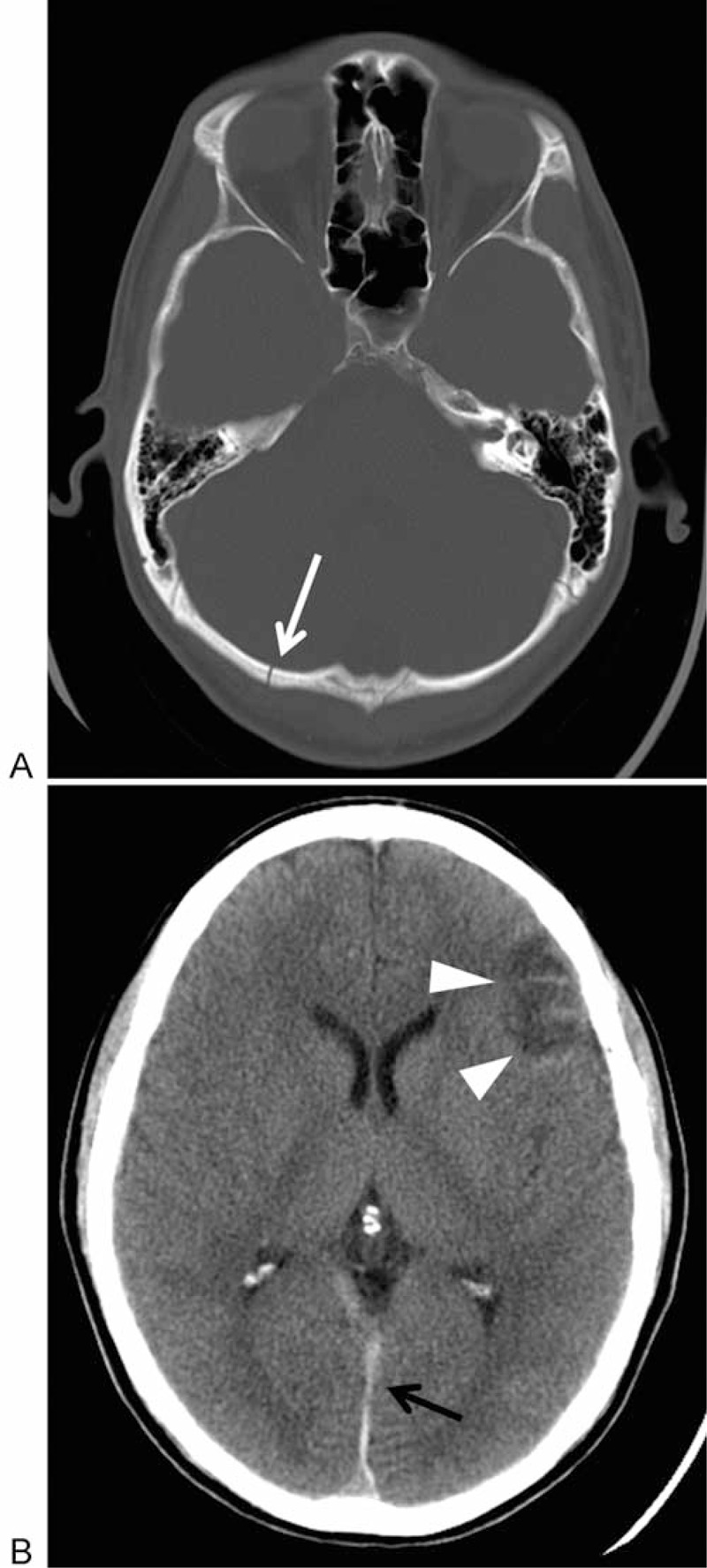
CT of the brain in a 23-year-old male with occipital bone fractures. He fell after alcohol intoxication. Four days later, he visited our ED with the chief complaint of headache and nausea. Axial CT shows fractures of the occipital bone (A, white arrow) and intracranial hemorrhage (B, black arrow) with brain contusion (arrow head).

Of the patients who presented to the ED over 24 hours after injury, the mean days to presentation in the sober group was greater than the intoxicated group. This may be because many elderly patients in the sober group had fractures with slowly worsening, protracted pain such as compression fractures or femoral neck fractures. However, interestingly, there were no sober patients with skull or cervical spine fractures who presented over 24 hours after sustaining fractures, while 22.0% of intoxicated patients with delayed presentation had these types of severe fractures (Figs. 1 and 2). Additionally, most head/neck fractures in intoxicated patients with delayed presentation were caused by low-energy mechanisms. This suggests that it is important to recognize the history of alcohol intake in the emergency setting. Also, emergency physicians need to maintain high clinical suspicions of skull and cervical spine fractures in patients who were intoxicated but sobered by the time of presentation to the ED.

### Fracture Locations

Intoxicated patients had a higher rate of head/neck fractures. On the other hand, they had a lower rate of extremity fractures than the sober group with statistical significance. This may have been due to the relatively high proportion of falls (see below) and altered balance/mechanics with intoxication leading to loss of protective reflexes.

When people fall, they usually outstretch their hands or legs to compensate. This protective mechanism of falling on an outstretched hand (FOOSH) can minimize the impact, sometimes resulting in forearm or hand fractures but absorbing energy that might have otherwise been transferred to more vital structures. In the intoxicated patients, the inability to compensate with a FOOSH mechanism may have resulted both in a lower incidence of limb injury and a greater incidence in cervical spine/head injury. Intoxicated patients also may have a propensity to fall from intermediate heights such as stairs or platforms because of their altered balance. Johnston et al^12^ reported a similar anatomic distribution of injuries in intoxicated patients, but our study includes a more detailed analysis of combined injury mechanisms.

### Injury Mechanism

Intoxicated patients had a greater incidence of falls from intermediate heights defined as those of less than 20 feet but greater than from standing. While this likely relates to the impaired balance intoxicated patients suffer, additionally the environmental opportunity for such falls is probably greater in people who are intoxicated and returning to home from after-work or after-school drinking because they generally use public transportation that requires navigation of platforms, escalators, and stairs.

In a related vein, a smaller proportion of intoxicated patients in our study population sustained fractures in motor vehicle accidents compared to sober patients. This may seem counterintuitive given the many recent studies indicating that alcohol intoxication increases the risk of traffic accidents and injury.^14,15^ However, since our hospital is located in a densely populated urban area, most people use the well-established public transportation and rarely drive cars, possibly explaining the lower numbers of automobile accidents in intoxicated patients. Further, Japan has strict laws to reduce alcohol-impaired driving,^16^ where the absolute number of traffic deaths fell from 11,451 in 1992 to 7358 in 2004, an average decrease of 3% to 4% per year^17^ following the enactment of such laws.

### Initial Radiologic Interpretation Accuracy

The rate of missed fractures by emergency physicians on initial interpretation but later identified by radiologists was 7% for both the intoxicated and sober groups. A previous study by Petinaux et al^13^ showed that about 3% of radiographs interpreted by emergency physicians were subsequently given a discrepant interpretation by radiologists, and the most common missed findings were fractures in their study. Our study showed a higher rate of discrepancies, but this rate is reasonable because we focused on only fractures.

### Limitations

This study has several limitations. First, we did not investigate the relationship between degree of alcohol intoxication and the severity of injuries, though many recent studies indicate that alcohol intoxication increases the risk of severe injury. Although the Injury Severity Score (ISS) is commonly used to measure the severity in trauma patients, we did not use this scoring system since our study was focused on fractures. Second, we did not use quantitative assessments of intoxication such as blood alcohol concentrations because alcohol testing is usually performed selectively in our hospital based on clinical suspicion and was not consistently available. Because we used radiologist interpretation as the gold standard to identify fracture cases, it may be possible that some cases were omitted because fractures could have been missed both by ED clinicians and radiologists. Third, this was a retrospective study conducted at a single institution. Our hospital is also a general hospital in an urban Japanese setting and the percentage of motorists is smaller than in rural areas. Therefore, our results might not be generalizable to rural populations. A multicenter study with a large number of patients will be needed in the future.

## CONCLUSION

While the same proportion of intoxicated patients presented more than 24 hours following injury, only intoxicated patients presented with craniofacial and cervical spinal fractures during this period. Alcohol-related injuries are more often associated with head/neck fractures but less extremity injuries, likely related to ineffective protective reflex mechanisms. Intoxicated patients fall more often from intermediate heights of <20 feet compared to sober patients. The rate of fractures missed by emergency physicians on radiographs and CT studies but later diagnosed by radiologists was the same in intoxicated and sober patients, with a miss rate of 7%.
